# The Development of a System for Elbow Joint Range of Motion Measurement Based on Image Recognition and Myoelectric Signals

**DOI:** 10.3390/life14121534

**Published:** 2024-11-22

**Authors:** Hsuan-Kai Kao, Yi-Chao Wu, Chi-Heng Lu, Xiu-Ling Hou, Tsair-Fwu Lee, Chiu-Ching Tuan

**Affiliations:** 1 Department of Orthopedic Surgery, Chang Gung Memorial Hospital at Linkou, Taoyuan 33305, Taiwan; samiyadon@cgmh.org.tw; 2 Bone and Joint Research Center, Chang Gung Memorial Hospital at Linkou, Taoyuan 33305, Taiwan; 3 College of Medicine, Chang Gung University, Taoyuan 33305, Taiwan; 4 Department of Electronic Engineering, National Yunlin University of Science and Technology, Yunlin 64030, Taiwan; alanwu@yuntech.edu.tw; 5 Department of Radiation Oncology, Chang Gung Memorial Hospital at Linkou, Taoyuan 33305, Taiwan; chiheng@cgmh.org.tw; 6 Department of Electronic Engineering, National Taipei University of Technology, Taipei 10608, Taiwan; abby2345345@gmail.com; 7 Medical Physics and Informatics Laboratory of Electronics Engineering, National Kaohsiung University of Science and Technology, Kaohsiung 80778, Taiwan; tflee@nkust.edu.tw; 8 Graduate Institute of Clinical Medicine, Kaohsiung Medical University, Kaohsiung 80778, Taiwan; 9 Department of Medical Imaging and Radiological Sciences, Kaohsiung Medical University, Kaohsiung 80778, Taiwan

**Keywords:** elbow joint range of motion measurement, myoelectric signal, image recognition, total harmonic distortion, rehabilitation

## Abstract

After a fracture, patients have reduced willingness to bend and extend their elbow joint due to pain, resulting in muscle atrophy, contracture, and stiffness around the elbow. Moreover, this may lead to progressive atrophy of the muscles around the elbow, resulting in permanent functional loss. Currently, a goniometer is used to measure the range of motion, ROM, to evaluate the recovery of the affected limb. However, the measurement process can cause measurement errors ranging from 4 to 5 degrees due to unskilled operation or inaccurate placement, leading to inaccurate judgments of the recovery of the affected limb. In addition, the current measurement methods do not include an assessment of muscle endurance. In this paper, the proposed device combines image recognition and a myoelectric signal sensor to measure the joint movement angle and muscle endurance. The movement angle of the elbow joint is measured using image recognition. Muscle endurance is measured using the myoelectric signal sensor. The measured data are transmitted to a cloud database via an app we have proposed to help medical staff track a patient’s recovery status. The average error value of static image recognition and verification is up to 0.84 degrees. The average error value of dynamic image recognition and verification is less than 1%. The average error of total harmonic distortion (THD) verified by the myoelectric signal sensor is less than ±3%. It was proven that our system could be applied to measuring elbow joint range of motion. Since this is pilot research, most of the measurement subjects are healthy people without dysfunction in arm movement, and it is difficult to observe differences in the measurement results. In the future, experiments will be conducted on patients with elbow fractures through the IRB. This is expected to achieve the effect of encouraging patients to be actively rehabilitated at home through their measurement data and images of their actions being displayed in real time using our cheap and compact device and app.

## 1. Introduction

Elbow fractures are usually caused by direct or indirect impacts, falls, sport strains, sprains, and dislocations [1]. After a fracture, the pain and discomfort in the affected area will cause the patient to have reduced willingness to bend and extend their elbow joint. Muscle atrophy, contracture, and stiffness may occur around the elbow. Hence, it is important to perform rehabilitation exercises for patients after surgery to prevent sticky and stiff joints, poor local blood circulation at the fracture site, and progressive atrophy of the muscles around the elbow. This can therefore reduce the probability of permanent loss of function in the elbow [2].

The standard for detecting normal limb joints is examination based on the range of motion, ROM [3]. A general goniometer is usually used to detect the range of angles of movement of a patient’s injured joint in clinical treatment by a physical therapist [4]. However, the error in the joint movement angle can be between 4 and 5 degrees due to the low operating proficiency of measurement personnel and inaccurate placement of goniometers [5].

A patient’s elbow fracture not only causes tearing, damage, and dislocation but also may affect the surrounding tissues in the affected part. Some mobility of the elbow may be lost after a fracture. V. Jones pointed out that rehabilitation exercises performed by patients after fractures could have a positive impact on these injuries [6].

In a study by Y. C. Chen et al., a system combining Microsoft Kinect devices with dynamic motion capture software called Vicon Nexus was used to detect the joint movement angles. To obtain the experimental results, eight infrared cameras, two Microsoft Kinect devices, and some infrared sensing devices were used to measure the range of the human body, the angles of joint movement, and the difference in joint movement speed for analysis by doctors and physical therapists. Since the overall cost of this system is too expensive and its parts need to be spread out evenly in the measured space, it is difficult to use at home [7].

In a study by K. Yamaura et al., electromagnetic sensors were placed onto a simulated forearm bone and a simulated upper arm bone, which were used to calculate five three-dimensional positions of bone landmarks. In calculating the vector relationship, the elbow joint’s flexion angle could be calculated based on the mathematical formula for the cosine of the supplementary angle [8].

In a study by F. Vauclair et al., a built-in app and a three-axis accelerometer were used to measure the angle of elbow joint movement [9]. It showed that the average error of flexion in joint activity is 6.4 degrees, the average error of the extension angle is 1.8 degrees, and the average error of the supination angle is 5.9 degrees. It showed that the mobile phone app could easily obtain the angle of joint movement. However, the mobile phone itself did not have the fixed arm of a universal goniometer. Hence, it was difficult to align the joint’s center of rotation, leading to an increase in errors in the measurement angle.

A study by Swe et al. focused on video surveillance systems for seniors living independently. The main concern was patients with chronic diseases and adults with reduced physical fitness, especially given falls among the elderly [10].

C.N. Phyo et al. proposed a smart HAR system by combining image processing and deep learning technology to automatically identify human daily activities using a depth sensor with information on the human skeleton. Furthermore, the use of the skeleton information was proven to be very promising due to the low computational cost and accurate results. In addition, it could be used without any restrictions on the environment and the domain structure [11].

A study by J. Shi et al. used instant ultrasound images and EMG signals to analyze variations in muscle thickness and fatigue levels. It showed that both the muscle variation signals and real-time ultrasound images could represent muscle fatigue status. However, the above equipment is not easy to obtain, and the ultrasonic equipment needs to be used by professionals. Therefore, it is not suitable for use at home [12].

Static stretching is widely used as a rehabilitation therapy to reduce muscle tension, which is used as an indicator of muscle fatigue and affects limb recovery. N. Okamura, etc., used the reaction force between the device they developed and the skin to obtain muscle stiffness data [13]. This developed device could be used to detect the stiffness of the superficial muscles of the human body and the degree of muscle fatigue. Since the human muscles are squeezed when using this device for measurement, this causes discomfort. Moreover, the properties of human muscles differ. Hence, the device’s parameters need to be optimized in a timely manner, in advance.

The status of the bicep muscles was detected by Y. Wang, etc., using a portable ultrasound system [14]. The results showed that both the muscle thickness and attenuation coefficients could assess the status of muscle fatigue. Since this ultrasonic equipment is expensive and it needs to be operated by professionals, it is not suitable for use at home.

Injury of the elbow not only limits the angle of joint movement but also affects the surrounding muscle tissue to cause stiffness, joint adhesion, and muscle weakness. Hence, a device for detecting the elbow joint’s activity angle and muscle endurance was proposed in this paper to address the above issues. The angle measurement errors caused by the existing gold-standard methods, namely the use of clinical mechanical goniometers, could be reduced to complete the muscle endurance data with the device we designed. In addition, auxiliary physical therapists could provide better rehabilitation treatment plans for patients when using the device we designed.

## 2. Materials and Methods

### 2.1. System Architecture

**Figure 1:**
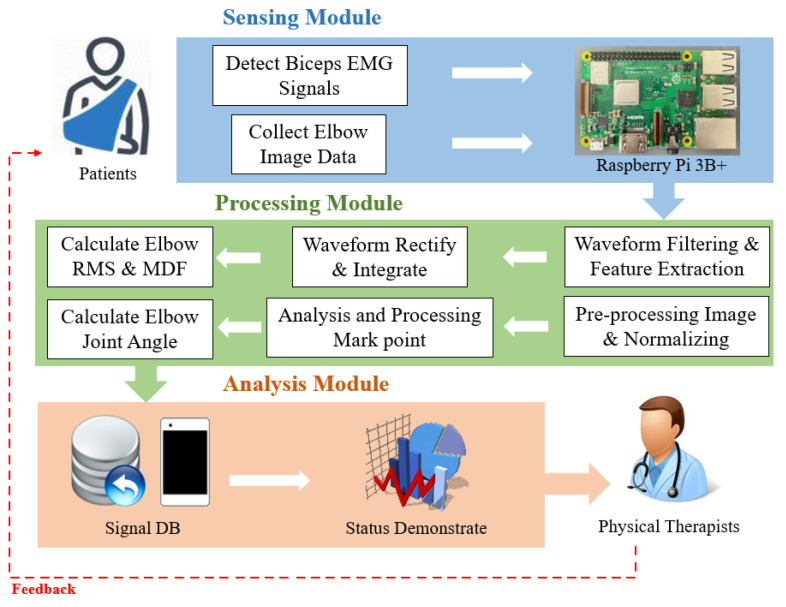
System architecture of elbow angle and muscle endurance sensing.

In our system, both an image recognition photography lens (Raspberry Pi Camera V2.1, launched by the Raspberry Pi company, Cambridge, UK) and an electromyography sensor (SparkFun MyoWare Muscle Sensor SEN-13723, Boulder, CO, USA) are used. The electromyographic signals of the biceps brachii muscles in the human arm could be captured using the myoelectric signal sensor. At the same time, marker points of the position of the elbow joint’s angle of movement were captured using Raspberry Pi Camera V2.1. Then, the data on the electromyographic signals were transferred to the Raspberry Pi development board directly. The joint activity angle value in vector coordinates was calculated using myoelectric signal processing, calculating the root mean square (RMS), the medium frequency (MDF), and the position of the marker points’ center coordinates in image recognition to monitor information on a subject’s muscle endurance and their presentation in terms of the elbow joint activity angle when performing active joint movements. The system architecture is shown in Figure 1.

In order to implement real-time dynamic and static object recognition and simultaneously process image and electromyographic signal data, the embedded system development board Raspberry Pi 4 Model B from Cambridge, UK, was used in this paper.

The image sensing module in Raspberry Pi Camera V2.1, made and launched by the Raspberry Pi company, Cambridge, UK, was used to sense images of the surrounding environment. The elbow joint movement angle could be recognized in real time. The sensing element in this module was the Sony IMX 219 PQ CMOS image sensor, which provides a high resolution and a high transmission rate to achieve real-time object recognition. Muscle endurance was detected using the SEN-13723 myoelectric signal sensor developed by SparkFun Electronics in Boulder, CO, USA, to measure the human body’s electromyography (EMG) signals using a non-invasive surface electrode method and output them. The output could be designed based on the user’s needs by selecting raw EMG signals from the RAW pin on the sensor that had not been processed or EMG signals from the SIG pin that had been noise-filtered and rectified. The power supply used was a lithium battery. The Raspberry Pi UPS expansion board, by the Raspberry Pi company in Cambridge, the UK, was used. The supply voltage was 3.7 V, and the capacity was 4000 mAh. Signals were transmitted by the I^2^C with an ADC (ADS1015) in the Raspberry Pi 4 Model B embedded system.

After the EMG signal is converted from analog into digital by the ADS1015, the image received by the CSI is transmitted to the embedded system through the built-in Wi-Fi module of Raspberry Pi 4 Model B for signal and image processing. Finally, the data are transmitted to the app through Wi-Fi.

### 2.2. The Measurement Position

**Figure 2:**
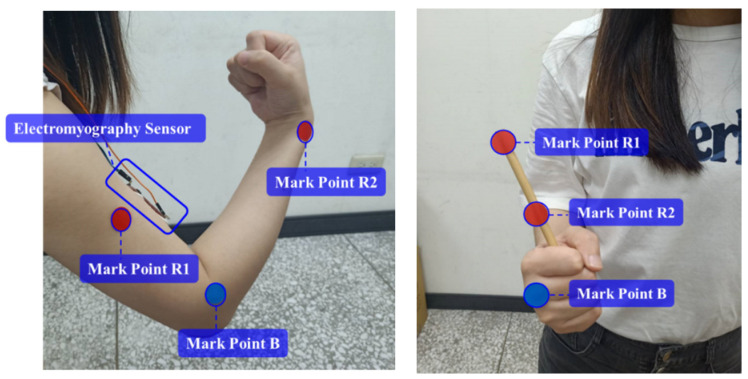
Position of elbow angle and muscle endurance sensing.

The muscle endurance and the swing angle of the joint are measured simultaneously using this device during human rehabilitation. The device is placed about 0.5 m away from the subject. At the same time, the myoelectric signal sensing module is stuck atop the subject’s arm on the bicep muscle belly. The myoelectric signal sensing module also needed to be parallel with the direction of the muscle fibers [15], as shown in Figure 2.

### 2.3. Joint Angle Image Recognition

OpenCV (v4.10.0) (Open Source Computer Vision Library) and Python (v3.13.0) were used for image recognition. OpenCV’s Hough Circle Transform function was used to extract the positions of the centers of the marker points in xy-plane coordinates on the human body. The positions of the marker points were obtained by calculating the radial distance of a circle [16].

Color, saturation, and HSV (hue, saturation, and value) were used to capture the color of the marker points to determine whether the marker points were the required detection object or not.

Finally, the marker point position obtained was calculated according to a mathematical formula for vector coordinates to obtain the angle of a marker point’s direction vector to obtain the joint activity angle *θ*. The HSV value in the OpenCV function library is closest to the colors seen by the human eye, so HSV is often used as the benchmark for marker point color space detection.

A color space is defined based on three basic attributes: value, hue, and saturation [17]. Therefore, it is necessary to convert the RGB into the HSV color space through the OpenCV function. The conversion formulas are as shown in (1) for *H*, in (2) for *S*, and in (3) for *V*.

(1) $$H=\left\{\begin{array}{c} \frac{60\times \left(G-B\right)}{\left(V-\min\left(R,G,B\right)\right)},\mathrm{if}\;V=R\\ 120+\frac{60\times \left(B-R\right)}{\left(V-\min\left(R,G,B\right)\right)},\mathrm{if}\;V=G\\ \;\;\;\;240+\frac{60\times \left(R-G\right)}{\left(V-\min\left(R,G,B\right)\right)},\mathrm{if}\;V=B\;\;\;\;\\ \;\;\;\;\;\;\;\;\;0,\mathrm{if}\;R=G=B \end{array}\right\}$$

(2) $$S=\left\{\;\begin{array}{c} \frac{\left(V-\min\left(R,G,B\right)\right)}{V},\mathrm{if}\;V\neq 0\\ \;\;0,\mathrm{otherwise} \end{array}\right\}$$

(3) $$V=\max\left(R,G,B\right)$$

The joint angle is calculated by using vector coordinates. For example, the three coordinate points are A(*x*1,*y*1), B(*x*2,*y*2), and C(*x*3,*y*3). The coordinate position of B is the starting point. $\vec{v_{1}}$ is the direction from B to A, and $\vec{v_{2}}$ is the direction from B to C, as in (4). Finally, the angle θ between two vectors based on known vectors was calculated by using the inner product Formula (5), where θ was the angle between two vectors.

(4) $$\vec{v1}=\left(x1-x2,y1-y2\right)\;\vec{v2}=\left(x3-x2,y3-y2\right)$$

(5) $$\theta =\cos^{-1}\left(\frac{\vec{v_{1}}\cdot \vec{v_{2}}}{\left|\vec{v_{1}}\right|\left|\vec{v_{2}}\right|}\right)$$

### 2.4. Muscle Fatigue Signal Processing

Electromyography, EMG, is a medical technology for evaluating the physiological signals of human muscles. EMG can be divided into time domain signals and frequency domain signals. Time domain signals are defined as the force exerted by human muscles. Frequency domain signals are defined as the excitation frequency of the motor units of human muscle [18].

In this paper, EMG corresponds to the physiological signals of human muscle contraction measured using the myoelectric signal sensor. After conversion through an analog-to-digital conversion module, such as the ADS1015, EMG signals are transmitted to Raspberry Pi 4 Model B for signal processing. Physiological signals are processed through filtering and rectification. Then, the root mean square (RMS) of the time domain signal and the median frequency (MDF) of the frequency domain signal in EMG are calculated to obtain muscle endurance information through joint analysis of spectra and amplitude (JASA) to measure the electromyographic signals.

EMG signals usually fall between 10 Hz and 500 Hz [19]. The sampling frequency needs to be higher than 1000 Hz. Due to the limitations of the experimental hardware equipment and according to the Nyquist theorem, a sampling frequency of 1600 Hz was selected in this paper. The values of myoelectric signals may be positive and negative. In addition, the negative signals may not completely mirror the positive signals. In order to prevent negative signal values from being filtered out in incomplete signal distortion, full-wave rectification was used to retain the negative part of the EMG signals and convert all negative signals into positive signals to present the original EMG signals completely [20].

The electromyographic signals measured using the electromyographic signal sensor are filtered and rectified. The root mean square, RMS, can be obtained based on the time domain signal of the electromyographic signal, where the RMS value is the amplitude of the electromyographic signal [21]. After calculating the total slope, the trends in the changes in muscle endurance can be observed [22]. The RMS is then converted into a frequency domain value through power spectral density (PSD) based on the RMS value every second. The frequency domain value obtained is then calculated and presented using the median frequency (MDF) [23]. The trend in the changes in the total frequency slope thus can be obtained to obtain the muscle endurance information.

JASA is used to measure the changes in the amplitude and spectra of physiological electrical signals from specific muscle positions through surface electromyography to determine whether the muscle is in a fatigue state at the same time [24].

### 2.5. User Interface and Cloud Storage

The user interface in this paper is designed based on the Android operating system development platform. The current time, measured time, current angle, and maximal current angle are displayed in the app we designed. The pronation/supination angle of the elbow joint is divided into two levels based on the average elbow pronation/supination angle of ±80 degrees for adults [25].

**Figure 3:**
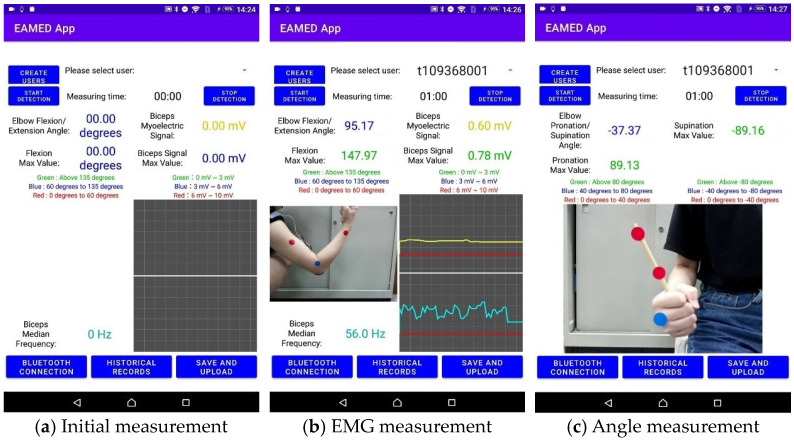
The app designed in this paper.

The measured data are displayed and stored in the SQLite database in real time for easy viewing and analysis. Users can click the “Save and Upload” button and select a date range so that medical staff can observe data remotely. The app will automatically save the data into an Excel file and upload it to the Firebase cloud database system, as shown in Figure 3.

### 2.6. Experimental Objects and Conditions

The environment of the measurement experiment was an indoor environment. The elbow joint angle of movement and the active rehabilitation exercises were communicated to the user before the measuring. The measured angle needed to be maintained for 3 s to confirm that the device could collect a stable amount of data.

Taking into account the complexity and uniformity of the overall experiment, the subject’s right elbow was used as the measurement site to explore the joint movement angle and related changes in the myoelectric signal measurement process. In this paper, the measurers were mainly the author and co-authors [26]. After the measurement results are analyzed and the system is optimized, an application to the IRB will be made based on clinical needs.

## 3. Results

### 3.1. Static Angle Verification

**Figure 4:**
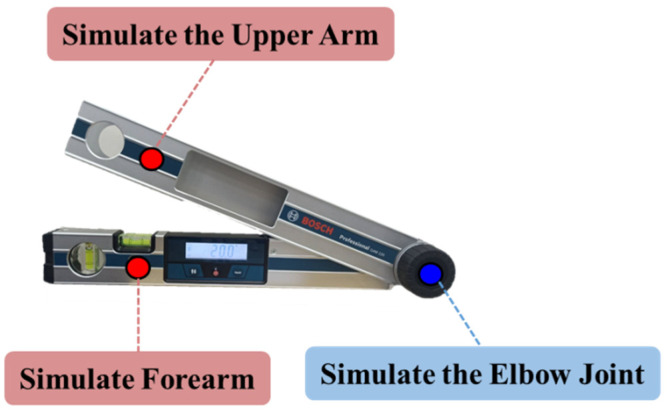
Static angle verification.

The reference angle was measured using a BOSCH GAM 220 (Bosch, Stuttgart, Germany) digital angle measuring instrument in this paper. At the same time, a high-resolution Nikon D7500 (Nikon Corporation, Tokyo, Japan) camera was used to detect the marker point above the digital angle measuring instrument to verify the accuracy of the Raspberry Pi camera in our system. The captured image was transferred to Raspberry Pi 4 Model B for marker point analysis and angle calculation to calculate the numerical error of the angle measured using both our device and the Nikon D7500 camera, as shown in Figure 4.

**Figure 5:**
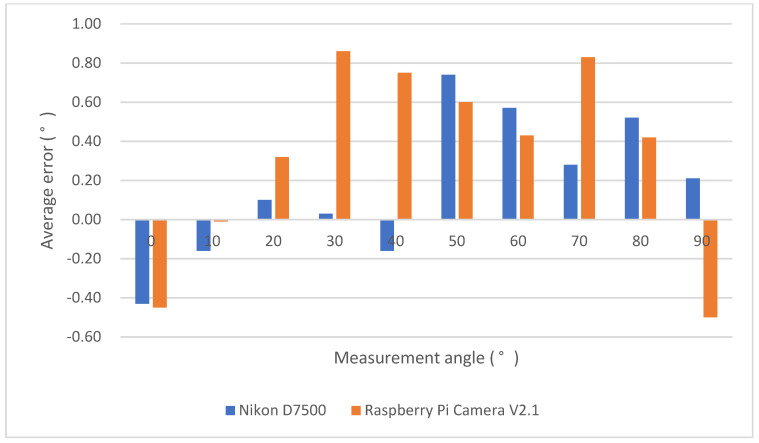
Average error of static angle verification experiment.

**Figure 6:**
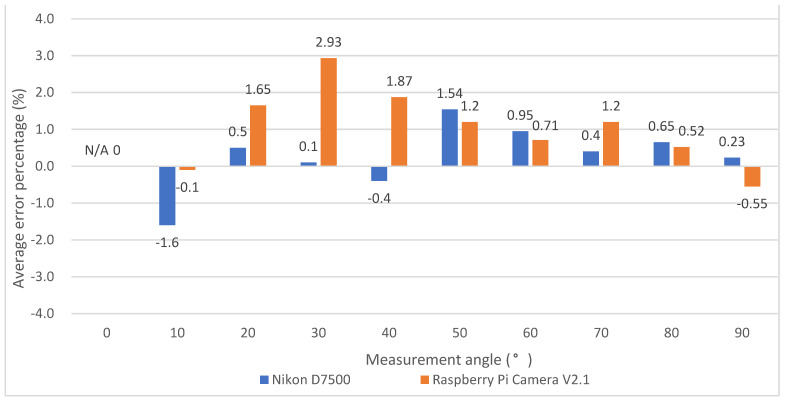
Average error percentage in static angle verification experiment. N/A is denoted when the static angle could not be detected by the NIKON D7500, while the measurement angle is 0 degrees.

The average error value and the average error percentage of the experimental results are shown in Figure 5 and Figure 6. In Figure 6, N/A denotes the static angle not being detected by the Nikon D7500 when the measurement angle was 0 degree. When the maximum angle error is 30 degrees in our device, the average error falls at 0.88 degrees, and the average error percentage is 2.93%. When the maximum angle error is 50 degrees in the Nikon D7500, the average error falls at 0.77 degrees, and the average error percentage is 1.54%. According to the clinically acceptable error value of ±5 degrees [27], it was proven that the angles measured by our device and the Nikon D7500 camera all had a certain degree of accuracy in the static verification experimental results.

### 3.2. Myoelectric Signal Verification

JASA (v1.2.8) was used to measure the changes in the amplitude and spectra of physiological electrical signals from specific muscle positions through surface electromyography to determine whether the muscle is in a fatigue state at the same time. An AFG-2225 signal generator was used for the output of the reference signal by receiving the original myoelectric signal through two sets of SEN-13723 myoelectric signal sensors. The processing signal from the AFG-2225 signal generator was transmitted to Raspberry Pi 4 Model B for numerical calculations, such as filtering and rectification.

According to the standards set by the American Electrical and Electronics Association IEEE 519-1992 [28], the maximum single harmonic of the voltage’s total harmonic distortion (total harmonic distortion, THD) cannot be higher than 3% of the fundamental wave in medical electronic equipment. This standard was used as the basis for the accuracy and reliability of the device in this paper.

The experimental design outputs a specific frequency and a sine waveform to two sets of myoelectric signal sensors. The original EMG signal is used for data acquisition and output, and the original EMG signal is used to calculate the total harmonic distortion THD using MATLAB (R2021a).

The maximum, minimum, average, and average error of the amplitude was measured at different frequencies of 1.5 mV, 3.5 mV, and 5.5 mV, as listed in Table 1. This showed that the average error of the sine wave amplitude did not exceed ±0.05 mV, and the average error percentage was within the acceptable range of ±3%.

### 3.3. Dynamic Signal Verification

**Figure 7:**
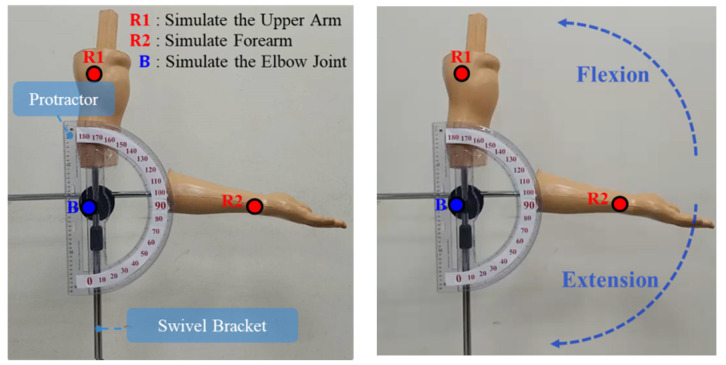
Dynamic identification and verification of joint flexion/extension angles.

In the dynamic experiment verifying simulated arm flexion and extension, a rotating stand was used, onto which the simulated arm was placed as the basis for the swing of the human elbow, as shown in Figure 7. The rotation axis used was the center point of the elbow joint, and the flexion and extension movements of the arm were simulated by swinging to verify the accuracy of the device in a dynamic environment.

The maximum standard deviation is ±0.40 degrees when the flexion/extension angle is from 120 degrees to 60 degrees. The maximum average error is +0.60 degrees when the flexion/extension angle is from 140 degrees to 40 degrees, as given in Table 2.

**Figure 8:**
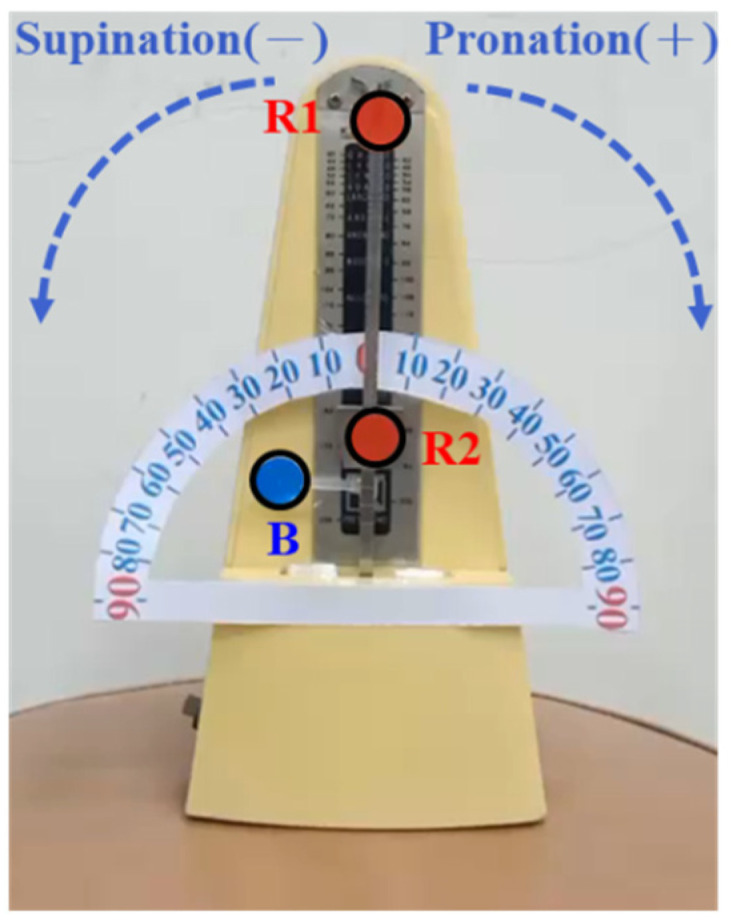
Dynamic identification and verification of joint pronation/supination angle.

In the dynamic simulation verifying arm pronation and supination, the swing mode is positive, and the offset angle of a clockwise swing is a positive pronation angle. The offset angle of a counterclockwise metronome swing is a negative supination angle. The average simulated angle value is ±60 degrees. R1 refers to the simulation involving holding the top of a pencil, R2 refers to the simulation involving holding the end of a pencil, and B refers to the simulation involving the knuckles of the mid-phalanges, as shown in Figure 8. In Table 3, it is shown that the maximum standard deviation of the experimental results is ±0.77 degrees when the pronation angle is 30 degrees. The maximum error value is −0.91 degrees at 50 degrees. The standard deviation of the supination angle is ±0.47 degrees at 60 degrees. The standard deviation of the supination angle is +0.97 degrees when the maximum error value is 40 degrees. Regardless of the pronation angle or the supination angle, the average error value is within 1 degree. It is proven that our device can be used to measure the elbow joint’s range of motion [27].

In the study by Y. Jeong, etc., 352 healthy subjects were measured [29]. The angle of human joint movement may be affected by factors such as age, BMI, medical history, gender, occupation, and living habits. For users aged 18 to 29, their average flexion angle was 146 degrees. For users aged 50 to 59, their average flexion angle was 143 degrees.

**Figure 9:**
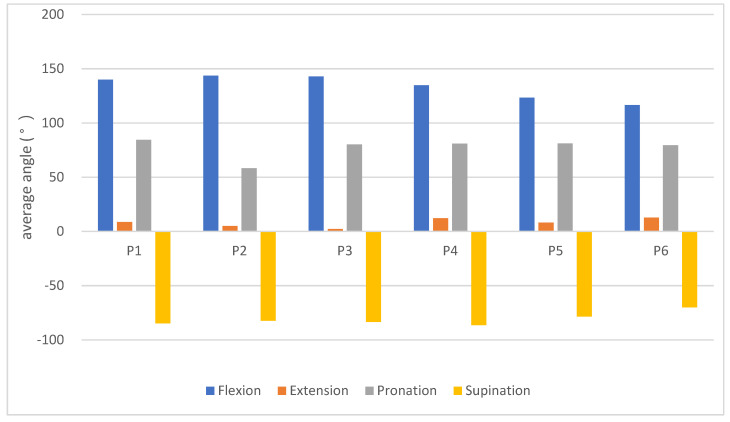
Measurements during active rehabilitation exercises of the elbow joint.

In research by D. W. Golden, etc., an increase in BMI may be caused by excess body fat that limits joint movement [30,31]. It also may be affected to a certain extent by changes in muscle mass. Restricted joint movement angles for a long time may cause stiffness or adhesion of the muscle tissue around the joints. Stickiness of the muscle tissue and loss of muscle fiber tissue may lead to insufficient muscle strength and endurance [2]. The experimental results on measurements of six subjects performing active elbow joint rehabilitation exercises are shown in Figure 9.

The average flexion angle of subjects P5 and P6 was more than 10 degrees lower than that of the other subjects. The average stretch angle value of all the subjects was between 0 and 13 degrees. The average extension angle of subjects P4 and P6 was higher than that of the other subjects.

According to the literature, the angle of movement of the elbow joint required for daily life is between 30 degrees of extension and 130 degrees of flexion. The angle of pronation and supination is about 50 degrees. Unless there are special angles required for large movements to stretch and bend, the above joint movement angles can be applied for normal life functions [30].

**Figure 10:**
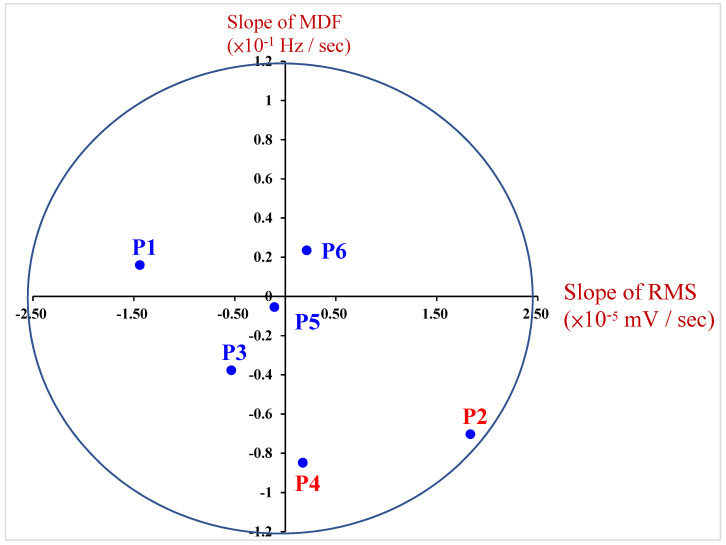
Results of measurements of active elbow joint rehabilitation exercises.

Based on amplitude–spectrum joint analysis (JASA) to observe the human body’s flexion/extension movements during active rehabilitation exercises, we analyzed the information on muscle endurance in the human biceps brachii. A comprehensive analysis was conducted based on the relevant parameters that have an impact on human muscle endurance, as shown in Figure 10.

## 4. Discussion

Only the average pronation angle of P2 was lower than that of the other subjects, and the difference in their average angle of supination exceeded 20 degrees. The average pronation angle of P6 was lower than that of the other subjects, with a difference in the angle of 10 degrees.

Although P5 had a history of elbow fracture, the data on P5 showed that only their flexion angle was relatively limited, and they could maintain the functions of daily life in the experiment according to the other angles. On the other hand, P6 did not have a history of elbow fracture, but his flexion and extension angles were lower than those of the other subjects. Their elbow joint angles were limited, and the supination angle was particularly low.

In [30], it was shown that the number of push-ups performed by the human body within 1 min can be used as a criterion for judging muscle endurance. Push-ups needed to be performed in standard form by male subjects, and push-ups needed to be performed in modified form by female subjects. A simple classification of muscle endurance was carried out based on the number of times push-ups could be performed. Six subjects were required to perform push-up training within 1 min.

The status of biceps brachii muscle fatigue in P2 and P4 could be measured after they performed the active rehabilitation exercises. Since the BMI of P2 and P4 was under 18.5 in both, with no habits of exercising, this may have led to insufficient muscle endurance. Although P5 had a history of elbow fractures, their muscle endurance was slightly better than that of P2 and P4. This was caused by P5 having the habit of exercising for effective maintenance of muscle endurance.

## 5. Conclusions

A measuring device for muscle endurance and the angle of elbow joint movement was developed based on image recognition and myoelectric signal sensors in this paper. From the experimental results using both Raspberry Pi Camera V2.1 and the Nikon D7500, the average error in the Nikon D7500 was up to 0.77 degrees compared with the standard digital angle measuring instrument. The average maximum error percentage was −1.60%. The average error in Raspberry Pi Camera V2.1 was up to 0.84 degrees compared with the standard digital angle measuring instrument. The average maximum error percentage was 2.93%. The error between the above two devices did not exceed ±3%.

Based on pasting marker points above the simulated arm (flexion/extension) and the metronome (pronation/supination), in static verification and in measuring the angle value during back-and-forth swinging in the device we developed, the highest average error of the experimental results was +0.60 degrees and +0.97 degrees, respectively. The maximum standard deviation was ±0.77 degrees, which was less than the acceptable range of 1%.

The average THD in both the specific amplitude and frequency generated from the signal generator was less than 3% for two different sets of myoelectric signal sensors. The average error value was less than ±0.05 mV and the average error was less than ±3% based on using the specific waveform and the specific frequency to verify static amplitude. This proves that the system device has a certain accuracy.

The elbow’s angle of flexion was slightly limited and the muscle endurance measured based on the electromyographic signal was decreased in the results for P5, with a history of elbow fracture and a slightly higher BMI. Muscle fatigue was displayed in both P2 and P4 through limited angles of joint movement. This may have been caused by their lower BMI, lack of habits of exercising, and limited angles of elbow joint movement.

Among all the subjects, the limitation in the joint movement angles in P6 was much higher than that in others since his BMI and age were higher than those of the others. However, the muscle strength of P6 is still increasing. It was shown that the level of muscle endurance in P6 is moderate. The preliminary assessment shows that although the subject is slightly older, his muscular endurance may be maintained at a moderate level due to his usual exercise habits.

In this paper, the system developed was in the pilot stage, and most of the experimental subjects were healthy without dysfunction in arm movements. Hence, it is difficult to observe differences in the measurement results. After the system is completed, experiments could be conducted on patients with elbow fractures through a hospital in human trials, according to an IRB. The effectiveness of rehabilitation assistance could be observed based on comparing subjects with normal elbow movement and elbow fractures. In this paper, we aimed to develop a low-price and compact-size device coupled with a self-developed app to display measured data, images, and actions in real time to encourage patients to be rehabilitated at home effectively.

Since the measurement system for the range of elbow joint motion based on image recognition and myoelectric signals proposed in this paper was part of pilot research, the experiments were conducted on six healthy subjects by the authors in this paper, without dysfunction in arm movement. Hence, it may be difficult to observe differences in the measurement results. To consider diversity in the experiments, the hospital will apply for a human trial (to the IRB) involving elbow fractures in the future. Depending on the IRB, the effectiveness of rehabilitation assistance between participants with elbow fractures and participants without elbow fractures could be evaluated. Moreover, patients could be encouraged to be rehabilitated at home effectively using the measurement system for elbow joint range of motion we have proposed at a low price and with a compact size, coupled with the self-developed app to display the accurate measured data, images, and actions in real time. The measured data are also stored in a cloud database via the self-developed app to be tracked by patients and medical staff safely.

## Figures and Tables

**Table 1:** Experimental results of static verification at 1.5 mV, 3.5 mV, and 5.5 mV.

Frequency (Hz)	Amplitude (mV)	Max Value (mV)	Min Value (mV)	Average Value (mV)	Average Error (mV)	Average Error Percentage (%)
50	1.5	1.547	1.431	1.508	+0.008	0.53
	3.5	3.563	3.420	3.498	−0.002	−0.06
	5.5	5.596	5.405	5.504	+0.004	0.07
100	1.5	1.559	1.263	1.469	−0.031	−2.07
	3.5	3.597	3.376	3.480	−0.020	−0.57
	5.5	5.592	5.395	5.459	−0.041	−0.75
150	1.5	1.591	1.343	1.494	−0.006	−0.40
	3.5	3.590	3.330	3.494	−0.006	−0.17
	5.5	5.579	5.330	5.495	−0.005	−0.09
200	1.5	1.598	1.340	1.475	−0.025	−1.67
	3.5	3.596	3.294	3.473	−0.027	−0.77
	5.5	5.588	5.256	5.473	−0.027	−0.49
250	1.5	1.596	1.364	1.487	−0.013	−0.87
	3.5	3.598	3.345	3.482	−0.018	−0.51
	5.5	5.597	5.317	5.484	−0.016	−0.29

**Table 2:** Joint activity angle in dynamic flexion and extension measurements.

Measuring Angle (A) (Degree)	Average Angle with Standard Deviation (B) (Degree)		Average Error (C = B − A) (Degree)	
	Flexion	Extension	Flexion	Extension
140 to 40	140.60 ± 0.15	39.90 ± 0.39	+0.60	−0.10
130 to 50	130.39 ± 0.28	49.89 ± 0.33	+0.39	−0.11
120 to 60	120.20 ± 0.40	59.82 ± 0.23	+0.20	−0.18
110 to 70	110.43 ± 0.20	70.34 ± 0.17	+0.43	+0.34
100 to 80	100.46 ± 0.37	80.16 ± 0.37	+0.46	+0.16

**Table 3:** Dynamic pronation and supination measurements of joint movement angles.

Measuring Angle (A) (Degree)	Average Angle with Standard Deviation (B) (Degree)		Average Error (C = B − A) (Degree)	
	Supination (+)	Pronation (−)	Supination (+)	Pronation (−)
±10	10.04 ± 0.33	−9.75 ± 0.15	+0.04	+0.25
±20	20.03 ± 0.16	−19.09 ± 0.21	+0.03	+0.91
±30	29.87 ± 0.77	−29.96 ± 0.21	−0.13	+0.04
±40	40.89 ± 0.19	−39.03 ± 0.17	+0.89	+0.97
±50	49.09 ± 0.38	−49.09 ± 0.22	−0.91	+0.91
±60	60.26 ± 0.22	−59.38 ± 0.47	+0.26	+0.62

## Data Availability

The data are contained within the article.
